# Persistent cell-associated HIV-1 RNA in virally suppressed individuals on INSTI-based ART

**DOI:** 10.1016/j.jve.2025.100609

**Published:** 2025-09-20

**Authors:** Kazuo Suzuki, Lucy Gold, Angelique Levert, Shannen Butterly, Emma Yoo, Takaomi Ishida, John Zaunders, Lucette A. Cysique, Bruce J. Brew

**Affiliations:** a St Vincent's Centre for Applied Medical Research, NSW State Reference Laboratory for HIV, Sydney, Australia; b St Vincent's Clinical School, Faculty of Medicine, Sydney, New South Wales, Australia; c School of Medicine, University of Notre Dame Australia, Australia; d Denka Co. Ltd, Tokyo, Japan; e UNSW Psychology, Sydney, NSW, Australia; f Departments of Neurology and Immunology and Peter Duncan Neurosciences Unit, St Vincent's Hospital, University of New South Wales and University of Notre Dame Sydney, Australia

**Keywords:** Cell-associated HIV-1 transcripts, Viral reservoir, INSTI, Cure research

## Abstract

Integrase strand transfer inhibitors (INSTIs) are the cornerstone of modern antiretroviral therapy (ART), achieving durable plasma HIV-1 suppression in most people living with HIV (PLWH). Previous comparisons of INSTI- and non-INSTI-based regimens have largely focused on HIV reservoir proviral assessments— typically total HIV DNA —without assessing reservoir activity. In this first functional comparison, we measured cell-associated (CA) short HIV-1 RNA transcripts, a marker of active transcription, alongside HIV-1 DNA in white blood cells from 92 virally suppressed individuals on INSTI-based (n = 73) or non-INSTI-based (n = 19) ART. CA short RNA transcripts were detected in all participants and HIV-1 DNA in 99 %, despite undetectable plasma viremia in >93 %. Individuals with prior “blips” — defined as a maximum of two episodes with 20–200 copies/mL plasma HIV-1 RNA over more than two years — had significantly higher CA RNA and HIV DNA than non-blip participants, confirming our previous findings. However, reservoir size and transcriptional activity did not differ significantly between INSTI and non-INSTI groups. These findings indicate that while INSTIs effectively block new integration events, they do not suppress ongoing transcription from the latent reservoir. Therapeutic strategies directly targeting HIV transcription should therefore be prioritized in cure-oriented research for PLWH on long-term suppressive ART.

## 1 Introduction

Potent antiretroviral therapy (ART) has transformed HIV infection into a manageable chronic condition. However, viral eradication remains elusive due to the persistence of long-lived infected cells harboring transcriptionally active proviruses. Even after years of sustained plasma HIV-1 suppression, these cells can produce viral RNA and proteins, contributing to chronic immune activation and tissue injury.^1^, ^2^, ^3^, ^4^

Multiple studies, including our own,^5^^,^^6^ have demonstrated that HIV-1 DNA persists in reservoir cells for prolonged periods despite suppressive ART. Techniques such as the Intact Proviral DNA Assay (IPDA),^7^ its enhanced versions optimized for HIV-1 subtypes B and C ^8^ and quadruplex qPCR (Q4PCR)^9^ confirm the stability of these reservoirs in peripheral blood. While most clinical studies have focused on reservoir proviral assessments through proviral DNA measurements, fewer have examined functional reservoir activity. Cell-associated HIV-1 RNA (CA-RNA) measurement provides a complementary perspective by quantifying transcriptional output from proviruses, regardless of their replication competence.^10^, ^11^, ^12^, ^13^ Elevated CA-RNA levels have been associated with adverse outcomes, including neurocognitive impairment and plasma viral “blips”,^6^^,^^14^^,^^15^ underscoring the importance of assessing both reservoir size and activity in people living with HIV (PLWH) on suppressive ART.

Integrase strand transfer inhibitors (INSTIs) are widely recommended as first-line ART and preferred agents in second-line therapy according to clinical guidelines from the World Health Organization (WHO) and the U.S. Department of Health and Human Services (DHHS).^16^^,^^17^ INSTI-based regimens, including dolutegravir (DTG) and bictegravir (BIC), are favored for their high potency, rapid viral suppression, high genetic barrier to resistance (especially DTG), and once-daily dosing convenience.^16^^,^^17^ Moreover, third-generation INSTIs with long-acting formulations and activity against viruses resistant to second-generation INSTIs are currently under clinical evaluation,^18^ positioning INSTIs as cornerstone ART agents for the future. Despite their virologic potency, the effect of INSTIs on HIV reservoir dynamics is poorly understood. Existing studies have focused mainly on proviral DNA,^19^ with limited assessment of transcriptional activity. Given their mechanism of action—blocking integration in the HIV life cycle—INSTIs could plausibly limit reservoir seeding and reduce ongoing transcription.

HIV DNA–based analyses have limitations for assessing reservoir dynamics, as most HIV-1 DNA in reservoirs is replication-incompetent.^20^^,^^21^ APOBEC3^22^, ^23^, ^24^ enzymes further restrict replication by introducing cytidine deamination, resulting in over 95 % defective proviruses.^20^ By contrast, CA-RNA analysis more directly reflects transcriptionally active reservoirs. Our “Double-R” assay targets the conserved R region in both the 5′ and 3′ long terminal repeats (LTRs), enabling detection of total spliced and unspliced HIV transcripts.^6^^,^^14^^,^^15^^,^^25^ While detection of short CA-RNA transcripts does not prove active replication, it indicates promoter-driven transcriptional activity.

In this study, we investigate the impact of INSTI-based ART regimens on HIV reservoir activity. Specifically, we assess levels of cell-associated HIV-1 short RNA transcripts and HIV-1 DNA in individuals receiving INSTI- versus non–INSTI-based regimens, with a focus on how viral blip history may modulate these effects.

## 2 Methods

### 2.1 Participants

This study was approved by the St Vincent's Hospital Human Research Ethics Committee (2019/ETH03527) and utilized standard-of-care samples submitted to the NSW State Reference Laboratory for HIV, St Vincent's Hospital, between 2017 and 2021 for plasma viral load (pVL) and CD4^+^ T-cell count monitoring. Demographic and HIV-1 disease characteristics, obtained from the St Vincent's Hospital patient database, are summarized in Table 1. The baseline dataset was divided into two groups: (i) INSTI-treated (n = 73) and (ii) non-INSTI-treated (n = 19). Viral blips were defined as elevated pVLs between 20 and 200 copies/mL during routine monitoring. For this study, the “Blip group” was defined as participants with a maximum of two such episodes over a period of more than two years, and the “Non-Blip group” as those without any viral blips during the same period.

**Table 1:** Baseline demographics and disease characteristics.

Demographics	INSTI	non-INSTI	p value
N (total 92)	73	19	
Age (Years), median (IQR)	56.0 (50–64)	54.0 (49–66)	ns (p = 0.96)
Sex: male, n (%)	72 (98.6 %)	17 (89.4 %)	∗ (p = 0.05)
***HIV Disease Characteristics, median (IQR)***			
HIV Infection Duration (years)	13 (6-15)	11 (7-16)	ns (p = 0.92)
Overall ART Duration (months)	57 (35–93)	74 (58–102)	ns (p = 0.13)
INSTI Duration (months)	39(17–61)	56 (31–102)^a^	ns (p = 0.12)
Blood CD4^+^ T-cell count	624 (446–878)	642 (550–784)	ns (p = 0.49)
Blood CD8^+^ T-cell count	783 (535–1090)	882 (518–1198)	ns (p = 0.54)
CD4/CD8 ratio	0.69 (0.44–1.00)	0.84 (0.73–1.16)	ns (p = 0.24)
**plasma Viral Load**	**0.0(0.0**–**0.0)**	**0.0(0.0**–**0.0)**	ns

a Non-INSTI Duration.

White blood cells (WBCs) were prepared from 6 ml of anticoagulated whole blood in ACD (acid citrate dextrose) tubes, as previously described.^6^ DNA and RNA were extracted using the Maxwell automated extraction platform (Promega, Madison, Wisconsin, USA), with the Maxwell RSC Buffy Coat DNA kit (Promega) and Maxwell RSC Simply R.NA Tissue kit (Promega), respectively, according to the manufacturer's protocol. WBCs were counted after red blood cell lysis using TC20 Automated Cell Counter (Bio-Rad, Hercules, California, USA), and these counts were used to normalize the HIV-1 DNA and RNA results as copy numbers/10^6^ cells.^6^

### 2.2 The double-R assay based on πCode end-point PCR assay

HIV DNA and short HIV-1 RNA transcripts were measured using the Double-R assay, as previously described.^6^^,^^14^^,^^15^^,^^25^ In brief, 92 individuals were analyzed (Table 1). HIV-1 copy number per sample was determined in duplicate using a standard curve generated from HIV-1 plasmid controls (6 points, 0.64–2000 copies/μL). Copy numbers were normalized to 10^6^ WBCs and reported as standardized units.

Primers and probes target the highly conserved “R” region present in both the 5′ and 3′ long terminal repeats (LTRs), enabling detection of total spliced and unspliced mRNA transcripts by one-step reverse transcriptase (RT)-PCR (Double-R assay; WO2018/045425 – PCT/AU2017/050974). For DNA PCR analysis, the RT step was omitted. Amplicons were detected using precision imaging of πCode (pi-Code) MicroDiscs (PlexBio) on the IntelliPlex platform (PlexBio), as described previously.^12^, ^13^, ^14^^,^^17^ Primer and probe designs are provided in the supplemental detaset of our previous publication. ^6^

### 2.3 Statistical analysis

We used GraphPad Prism v10 (GraphPad Software). For cohort demographics and disease characteristics, we conducted an a priori assessment of normality using the Anderson–Darling test, D'Agostino & Pearson test, Shapiro–Wilk test, and visual inspection of histograms. Continuous variables that were approximately normally distributed were compared using two-sample t-tests (unpaired *t*-test for Age), while non-normally distributed variables were compared using the Mann–Whitney *U* test (other continuous variables). Categorical variables were analyzed using χ^2^ tests (Sex). Exact p-values are reported in Table 1, with significant comparisons clearly annotated. Where appropriate, data are presented as medians (IQR).

HIV-1 short RNA transcriptional activity and HIV-1 DNA in WBCs was quantified as previously described.^14^^,^^15^ Standard curves were generated from serial dilutions of an HIV-1 plasmid using GraphPad Prism v10 (GraphPad Software). Comparisons of cell-associated (CA) short HIV-1 RNA transcript levels between INSTI and non-INSTI regimens were performed using the non-parametric Mann–Whitney *U* test. Associations between CA short HIV-1 RNA transcript levels and HIV-1 DNA levels were assessed using Pearson's correlation coefficient (r) with a two-tailed test.

## 3 Results

### 3.1 Detection of CA HIV-1 Short Transcripts in White Blood Cells (WBCs)

The demographics of the 92 individuals included in this study are summarized in Table 1. Participants were stratified into two subgroups according to ART regimen: INSTI-based and non-INSTI-based. The groups were comparable in age, duration of HIV infection, and ART duration, with similar CD4^+^ and CD8^+^ T-cell count ranges. All participants were on suppressive ART. These exploratory analyses confirm group comparability and do not influence the primary outcome analyses.

**Fig. 1:**
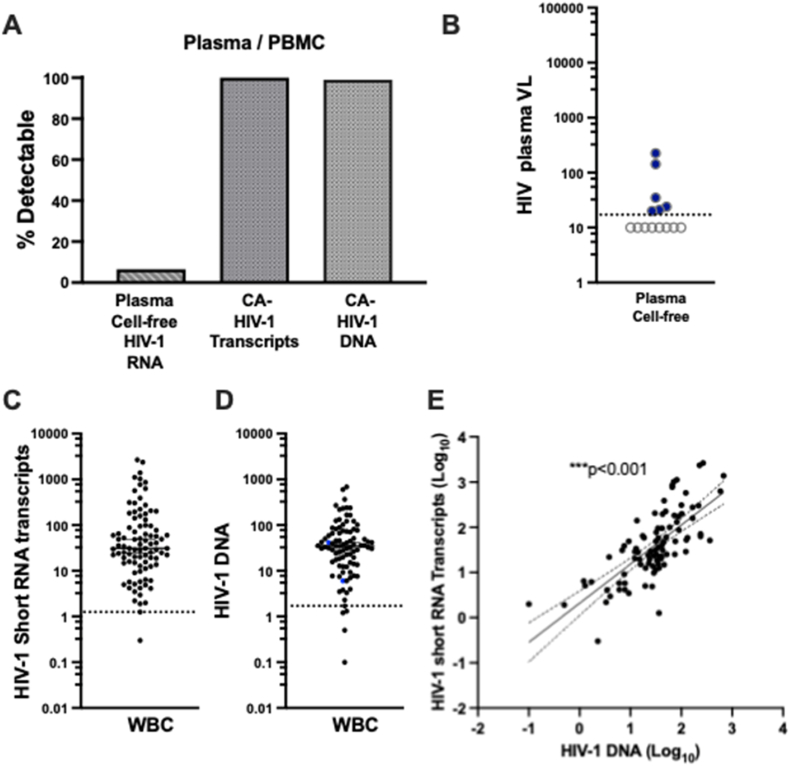
**Detection of CA short HIV-1 RNA transcripts and HIV-1 DNA in WBCs from PLWH with suppressed plasma viral load. (A)** Overview of plasma HIV-1 RNA detectability and levels of CA short HIV-1 RNA transcripts and HIV-1 DNA in circulating WBCs. Plasma viral load was undetectable in the majority of participants (93.4 %). In contrast, short HIV-1 RNA transcripts were detected in all individuals. **(B)** Plasma HIV-1 RNA levels across the cohort. The dotted line indicates the assay's limit of detection (LOD = 20 copies/mL). Values below the LOD were considered censored (also referred to as “below detection”), meaning that the exact RNA level is unknown but falls somewhere below 20 copies/mL. For visualization, these points are plotted at 10 copies/mL and shown as open circles. **Note**: This plotting convention does not reflect actual measured values; it is intended solely to indicate that the measurement was below the detection threshold. **(C)** Short HIV-1 RNA transcript levels expressed as copies per 10^6^ WBCs. The dotted line represents the LOD (2 copies/10^6^ cells). **(D)** HIV-1 DNA levels expressed as copies per 10^6^ WBCs. The dotted line represents the LOD (8 copies/10^6^ cells). **(E)** Significant correlation between the Short RNA transcripts and HIV-1 DNA levels. **Note**: The detection limits for CA short HIV-1 RNA transcripts and HIV-1 DNA were established using the latent HIV-infected cell line MO10-1. Lower-range data points are shown to reflect the strong assay signal relative to background noise.^15^**Note:** HIV short RNA transcripts and HIV DNA are shown as copy numbers normalized to 1 × 10^6^ WBCs (see Methods).

We quantified CA HIV-1 short RNA transcripts and HIV-1 DNA levels in total WBCs. Short RNA transcripts were detected in all 92 individuals (100 %), with a median of 31 copies per 10^6^ WBCs. HIV-1 DNA was also detected in 91 out of 92 individuals (99 %), with a median of 35 copies per 10^6^ WBCs (Fig. 1A–C, D). In contrast, plasma HIV-1 RNA was detectable in only 6.5 % of participants with a median below the detection threshold of standard assays (Fig. 1A and B). Importantly, a significant positive correlation (p < 0.001) was observed between CA HIV-1 short RNA transcripts and HIV-1 DNA levels, confirming our previous findings.^6^ This indicates that reservoir size (as measured by proviral DNA) and transcriptional activity (as measured by short HIV RNA transcripts) are closely linked in individuals on long-term ART. These results are consistent with our earlier reports,^6^^,^^14^^,^^15^ which demonstrated persistent HIV transcriptional activity in reservoir cells despite prolonged plasma viral suppression.

### 3.2 Impact of INSTI on CA short HIV RNA transcripts

**Fig. 2:**
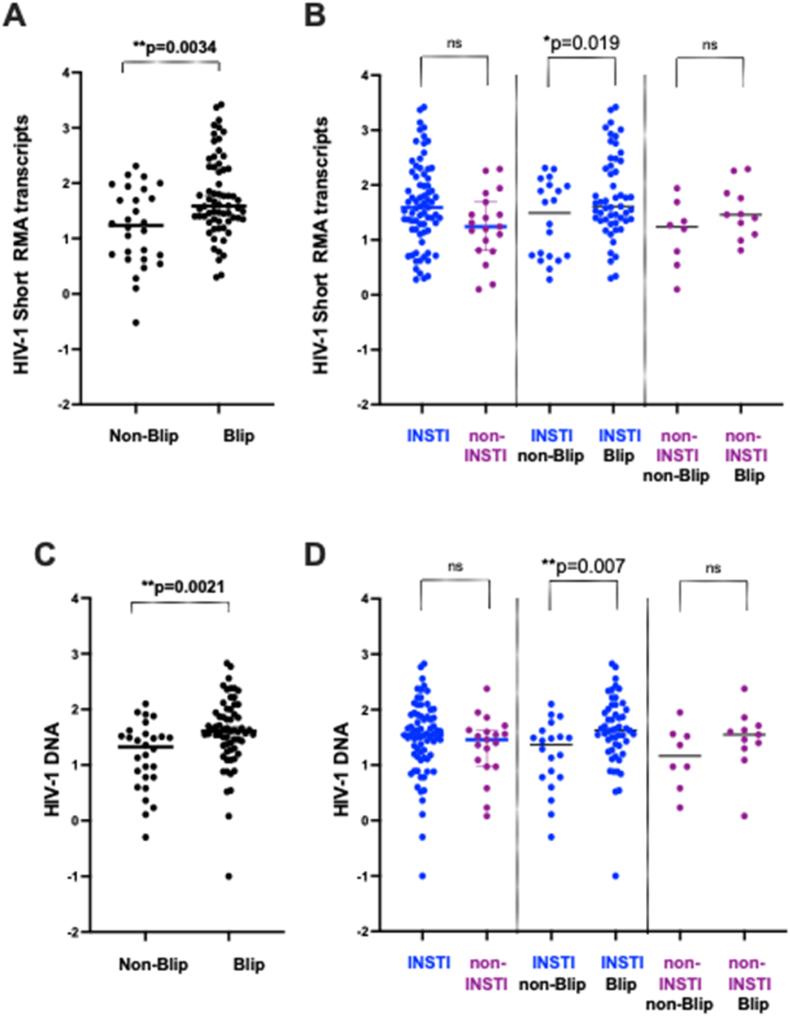
Impact of INSTI-based and non-INSTI based regimes on the CA short HIV-1 RNA transcripts and HIV-1 DNA in WBCs. (A) Comparison of CA short HIV-1 RNA transcript levels between individuals with and without prior plasma viral load viral blips. (B). CA short HIV-1 RNA transcript levels stratified by ART regimen (INSTI-based vs. non-INSTI-based) and viral blip history (non-blip vs. blip). (C). Comparison of HIV-1 DNA levels between individuals with and without prior viral blips. (D). HIV-1 DNA levels stratified by ART regimen (INSTI-based vs. non-INSTI-based) and viral blip history.

We focused our analysis of CA HIV-1 RNA transcripts on individuals who had experienced plasma viral blips. Building on our previous findings,^6^ we observed significantly higher levels of short transcripts in individuals with viral blips (defined as a maximum of two episodes of 20–200 copies/mL over a period longer than two years), with a median of 39 copies per 10^6^ WBCs, compared to the non-blip group, which had a median of 17 copies per 10^6^ WBCs (Fig. 2A).

Next, we examined whether INSTI-based ART regimens influenced levels of CA short transcripts. There was no significant difference in short RNA transcript levels between individuals receiving INSTI-based regimens and those on non-INSTI-based regimens (Fig. 2B, left two datasets). Importantly, we continued to observe a consistent effect of viral blip episodes: participants in the blip group had significantly higher CA short RNA levels compared to the non-blip group (Fig. 2B, middle two datasets; p = 0.019). Within the non-INSTI group, we observed a trend toward lower CA HIV RNA levels in the non-blip group compared with the blip group; however, these differences did not reach statistical significance, likely due to the small sample size in this subgroup (Fig. 2B, right two datasets).

### 3.3 Impact of INSTI on HIV DNA levels

We extended our analysis to examine HIV-1 DNA levels. As expected, individuals in the blip group had significantly higher HIV-1 DNA levels, with a median of 41 copies per 10^6^ WBCs, compared to the non-blip group, which had a median of 21 copies per 10^6^ WBCs (Fig. 2C). We then assessed whether INSTI-based ART regimens affected HIV-1 DNA levels. No significant difference was observed between individuals on INSTI-based regimens and those on non-INSTI-based regimens (Fig. 2D, left two datasets). Importantly, the effect of viral blips on HIV-1 DNA levels remained consistent: individuals with viral blips had significantly higher HIV DNA levels than those without (Fig. 2D, middle two datasets; p = 0.007). We observed a trend toward lower HIV DNA levels in the non-blip group, within the non-INSTI group compared with the blip group; however, these differences did not reach statistical significance, likely due to the small sample size in this subgroup (Fig. 2D, right two datasets).

Taken together, these findings demonstrate that both HIV short RNA transcripts and HIV-1 DNA levels are elevated in individuals with prior viral blips, even among those receiving INSTI-based regimens. This suggests that INSTI-based ART, while highly effective at plasma viral suppression, does not necessarily reduce transcriptional activity or reservoir size in circulating peripheral reservoir cells.

## 4 Discussion

This study demonstrates that INSTI-based ART regimens do not effectively reduce cell-associated (CA) HIV-1 RNA transcripts or HIV-1 DNA levels in viral reservoirs, despite being among the most potent and widely recommended first- and second-line treatment options. This limitation is likely due to the mechanism of action of INSTIs, which target the integration step of the HIV-1 life cycle,^26^ rather than directly suppressing transcriptional activity or eliminating reservoir cells.

Circulating peripheral blood reservoirs—the most accessible and clinically relevant source for monitoring HIV—have been shown to reflect reservoir activity occurring in various tissue compartments.^27^ As such, recent studies using advanced molecular tools, including the IPDA, RNA-IPDA, and Q4PCR, have uncovered ongoing transcriptional activity and persistence of intact proviruses, even in virally suppressed individuals.^7^, ^8^, ^9^, ^10^, ^11^, ^12^, ^13^^,^^28^, ^29^, ^30^

Importantly, our recent studies have linked elevated cell-associated HIV-1 RNA transcripts detected by the Double-R assay in reservoir cells to ongoing brain injury, as measured by in vivo proton magnetic resonance spectroscopy (^1^H-MRS)^31^**.** Additional evidence indicates that high levels of CA HIV RNA and DNA in cerebrospinal fluid (CSF) cells may contribute to HIV-associated neurocognitive disorders.^15^ Moreover, increased reservoir transcriptional activity has been associated with a higher incidence of plasma viral blip episodes.^14^^,^^15^ While CA-RNA short transcripts do not directly indicate productive HIV replication cycles, they serve as markers of any transcriptional activity from the HIV-1 promoter. Our previous data demonstrated a significant correlation between *ex vivo* short transcript levels in isolated CD4^+^ T cells and those measured after 3 days of *in vitro* culture with T cell activation^6^**,** suggesting that promoter activity captured by the Double-R assay reflects biologically relevant reservoir dynamics.

Additionally, individuals with extremally low levels of CA short RNA transcripts would be ideal candidates for cure strategies or analytical treatment interruptions.^28^^,^^32^^,^^33^ Monitoring CA short RNA transcripts should provide a sensitive biomarker of residual reservoir activity, particularly when plasma HIV-1 RNA remains below the detection threshold of standard clinical assays.

Our study has several limitations. The cohort comprised individuals with chronic HIV infection, and all in the INSTI group were receiving INSTIs as second-line therapy, preventing assessment of first-line INSTI effects. Future studies in treatment-naïve individuals may clarify whether INSTIs influence early reservoir seeding, and modelling efforts incorporating cellular turnover rates could better estimate reservoir dynamics. The non-INSTI group was relatively small (n = 19), reflecting the widespread adoption of INSTI-based ART both at St Vincent's Hospital and globally,^34^ making recruitment of non-INSTI patients challenging. Samples were standard-of-care specimens, and ART regimens were verified via hospital records prior to analysis, without selection bias. Larger cohorts will be needed to confirm our initial findings. Additionally, we did a post-hoc power analysis comparing the INSTI (n = 73) and non-INSTI (n = 19) groups, assuming a medium-to-large effect size (Cohen's d = 0.75) and α = 0.05, yielded a power of 0.82. This indicates an ∼82 % chance of detecting a true difference. Smaller effect sizes would require substantially larger cohorts to demonstrate statistical significance. Nevertheless, the absence of significant differences in our study remains informative, showing that CA HIV-1 RNA transcription persists irrespective of ART class under viral suppression.

Despite these limitations, our findings underscore a critical point: plasma viral suppression alone does not reflect full control of HIV replication or its long-term pathological consequences. Persistent transcription of short HIV-1 RNA transcripts within the circulating reservoir likely contributes to continued neuropathogenesis. This study highlights the complexity of reservoir maintenance and suggests that mechanisms beyond integration may contribute to persistent transcription in ART-suppressed individuals.

In conclusion, monitoring and targeting reservoir transcriptional activity may be essential for optimizing the clinical management of people living with HIV and for advancing future HIV cure strategies.^35^, ^36^, ^37^

## CRediT authorship contribution statement

**Kazuo Suzuki:** Writing – review & editing, Writing – original draft, Visualization, Validation, Supervision, Software, Resources, Project administration, Methodology, Investigation, Funding acquisition, Formal analysis, Data curation. **Lucy Gold:** Resources, Investigation. **Angelique Levert:** Validation, Supervision, Resources, Methodology. **Shannen Butterly:** Methodology, Investigation. **Emma Yoo:** Methodology, Investigation. **Takaomi Ishida:** Software, Resources. **John Zaunders:** Investigation. **Lucette A. Cysique:** Writing – review & editing, Validation, Supervision, Resources, Methodology, Conceptualization. **Bruce J. Brew:** Writing – review & editing, Validation, Supervision, Project administration, Funding acquisition, Conceptualization.

## Funding

This research was partly funded by a St Vincent's Clinic Foundation Resarch Grant and AMR transplational Grant awarded to KS, with additional support from NHMRC Grant ID 1105808 awarded to BJB.

## Declaration of competing interest

KS is listed as an inventor on patent WO2018/045425 (PCT/AU2017/050974), which covers an analytical method for quantifying HIV-1 short RNA transcripts; related equipment and reagents are available from PlexBio Inc. All other authors declare no competing interests.

## Data Availability

The full dataset supporting this study is available on Figshare (DOI: 10.6084/m9.figshare.30133486) https://doi.org/10.6084/m9.figshare.30133486.
